# ^68^Ga-Trivehexin: Current Status of αvβ6-Integrin Imaging and Perspectives

**DOI:** 10.3390/cancers17091504

**Published:** 2025-04-29

**Authors:** Luca Urso, Rebecca Napolitano, Giorgia Speltri, Murat Tuncel, Ilham Badrane, Licia Uccelli, Francesca Porto, Petra Martini, Alessandro Niorettini, Corrado Cittanti, Mirco Bartolomei, Alessandra Boschi

**Affiliations:** 1 Department of Translational Medicine, University of Ferrara, Via Luigi Borsari 46, 44121 Ferrara, Italy; luca.urso@unife.it (L.U.); ccl@unife.it (L.U.); francesca.porto@unife.it (F.P.); corrado.cittanti@unife.it (C.C.); 2 Nuclear Medicine Unit, Department of Onco-Haematology, Via Aldo Moro 8, 44124 Ferrara, Italy; m.bartolomei@ospfe.it; 3 Department of Environmental and Prevention Sciences, University of Ferrara, 44121 Ferrara, Italy; rebecca.napolitano@unife.it (R.N.); petra.martini@unife.it (P.M.); alessandro.niorettini@unife.it (A.N.); 4 Department of Chemical, Pharmaceutical and Agricultural Sciences, University of Ferrara, 44121 Ferrara, Italy; giorgia.speltri@unife.it (G.S.); alessandra.boschi@unife.it (A.B.); 5 Department of Nuclear Medicine, Hacettepe University, 06230 Ankara, Turkey; murat.tuncel@hacettepe.edu.tr

**Keywords:** [^68^Ga]Ga-Trivehexin, trivehexin, αvβ6-integrin, molecular imaging, precision oncology, PET

## Abstract

Background/Objectives: Molecular imaging, especially PET, has advanced significantly, shifting from metabolic radiotracers like 2-deoxy-2-[^18^F]fluoro-D-glucose [^18^F]FDG to target-specific probes. Among these, αvβ6-integrin has emerged as a promising target in cancer and non-cancer diseases. This review focuses on the radiochemical properties and initial clinical applications of the [^68^Ga]Ga-Trivehexin PET probe. Methods: The literature review on [^68^Ga]Ga-Trivehexin systematically evaluated both preclinical and clinical studies, with particular emphasis on its radiochemical characteristics and preliminary clinical applications, while highlighting advancements, associated challenges, and the potential for future developments in the field. Results: This study highlights the significant advancements achieved with [^68^Ga]Ga-Trivehexin in the field of molecular imaging. The optimized multimeric system has substantially enhanced the radiotracer’s pharmacokinetic properties, binding affinity, and selectivity for αvβ6 integrin, demonstrating up to an 18-fold improvement compared to previous monomeric tracers. The synthesis protocol has been refined to achieve high radiochemical purity (>95%), essential for safe clinical use. Preliminary clinical applications, particularly in head and neck cancer (HNC) and pancreatic ductal adenocarcinoma (PDAC), have shown promising results, with high detection rates and improved differential diagnosis compared to [^18^F]FDG. Furthermore, [^68^Ga]Ga-Trivehexin PET/CT has shown potential in non-oncological conditions, such as idiopathic pulmonary fibrosis (IPF) and primary hyperthyroidism, suggesting broader clinical applicability. Conclusions: [^68^Ga]Ga-Trivehexin is a promising PET probe for imaging αvβ6-integrin in cancers and non-oncological diseases like idiopathic pulmonary fibrosis (IPF) and primary hyperparathyroidism (PHP).

## Simple Summary

Significant advancements have been made in molecular imaging, particularly positron emission tomography (PET), which has evolved from using metabolic radiotracers like 2-deoxy-2-[^18^F]fluoro-D-glucose [^18^F]FDG to more targeted probes. Among these, αvβ6-integrin has emerged as a promising target for both oncological and non-oncological diseases. This review explores the radiochemical properties and initial clinical applications of the PET probe [^68^Ga]Ga-Trivehexin. Preclinical and clinical studies are discussed, focusing on its radiochemical characteristics and preliminary clinical uses, highlighting advancements, challenges, and future potential. The results show that the optimized multimeric system significantly improved pharmacokinetic properties, binding affinity, and selectivity for αvβ6-integrin, achieving up to an 18-fold enhancement compared to previous monomeric tracers. Preliminary clinical applications, particularly in head and neck cancer and pancreatic adenocarcinoma, have demonstrated promising detection rates and improved differential diagnosis compared to [^18^F]FDG. Additionally, [^68^Ga]Ga-Trivehexin has shown potential in non-oncological conditions such as idiopathic pulmonary fibrosis and primary hyperthyroidism. This review emphasizes the progress made in the design and application of [^68^Ga]Ga-Trivehexin, a highly promising PET tracer for both oncological and non-oncological imaging.

## 1. Introduction

Molecular imaging has had an impressive evolution in the last decade [1]. Considering positron emission tomography (PET) imaging, the first chapters of this evolution saw the widespread use of metabolic radiotracers. Among those, 2-deoxy-2-[^18^F]fluoro-D-glucose ([^18^F]FDG) has been and still is the most widely used PET radiotracer employed in daily clinical practice, with a growing multitude of oncologic and non-oncologic applications [2,3,4,5,6]. Besides [^18^F]FDG, numerous other metabolic radiotracers have been proposed and subsequently implemented in daily clinical practice, including radiolabeled choline and amino acidic radiotracers (i.e., [^18^F]F-Fluciclovine, [^18^F]F-Fluorodopa, [^11^C]C-methionine, and many others) [7,8,9,10]. Afterwards, the evolution of target-specific PET molecular probes has signaled a revolution in PET imaging. The first glance into the potentialities of cancer-specific molecular targets was offered by radiolabeled somatostatin analogs, who offered the first theranostic model in neuroendocrine neoplasms [11]. Afterwards, radiolabeled prostate-specific membrane antigen (PSMA) ligands have revolutionized the landscape of prostate cancer imaging and, more recently, therapy [12,13,14].

The investigation for new target-specific radiotracers is at the forefront of actual translational research, with plenty of new PET probes being explored [15,16,17]. Among those, radiotracers targeting the integrin protein family are among the most promising for clinical translation [18]. Integrins are a group of 24 transmembrane glycoproteins involved in cell adhesion. These proteins are composed of the dimerization of two subunits: α—which has 18 different isoforms—and β—counting eight variants. The different combinations of α and β subunits originate the integrin proteins, which are characterized by very different functions and expression throughout the human body. Of note, some integrin proteins are involved in carcinogenesis [19,20]. Among cancer-related integrins, αvβ3 has been the first under investigation since the early 1980s [21]. αvβ3-integrin is related to angiogenesis, both in physiological conditions (i.e., wound healing and embryonal development) and in solid tumor growth [22]. Of note, high-affinity antagonists of αvβ3-integrin—collectively known as RGD peptides—have been used to selectively target αvβ3-integrin [23]. Some of those have also been radiolabeled with radionuclides for PET or Single Photon Emission Tomography (SPECT) to map the in vivo distribution of αvβ3-integrin expression [24]. However, these radiotracers did not meet the initial high expectations. The average αvβ3-integrin expression in solid tumors was not high enough to emulate the performances of the other theranostic models currently used in daily clinical practice [22].

More recently, the researchers’ attention shifted towards αvβ6-integrin. Its expression is typical of epithelial cancer cells, while its levels are almost absent in normal tissues [22]. Overexpression of αvβ6-integrin is related to the transforming growth factor β (TGF-β) pathway and activates tumorigenesis [25]. Therefore, αvβ6-integrin is a suitable target for selective molecular imaging of epithelial carcinomas, in particular pancreatic cancer, head and neck carcinomas and colon cancer [26]. Moreover, since TGF-β is correlated to extracellular matrix (ECM) remodeling, αvβ6-integrin is also overexpressed in some non-oncological conditions, in particular idiopathic pulmonary fibrosis (IPF) [27].

Recently, [^68^Ga]Ga-Trivehexin has been proposed as a PET probe to map in vivo expression of αvβ6-integrin expression and has started its translational journey from preclinical to clinical experiences. In this review, we aim to provide an updated overview of the radiochemical features of this radiotracer. Moreover, initial clinical experiences with [^68^Ga]Ga-Trivehexin PET in oncological and non-oncological diseases will be discussed.

## 2. [^68^Ga]Ga-Trivehexin: A Radiochemical Insight

**Figure 1:**
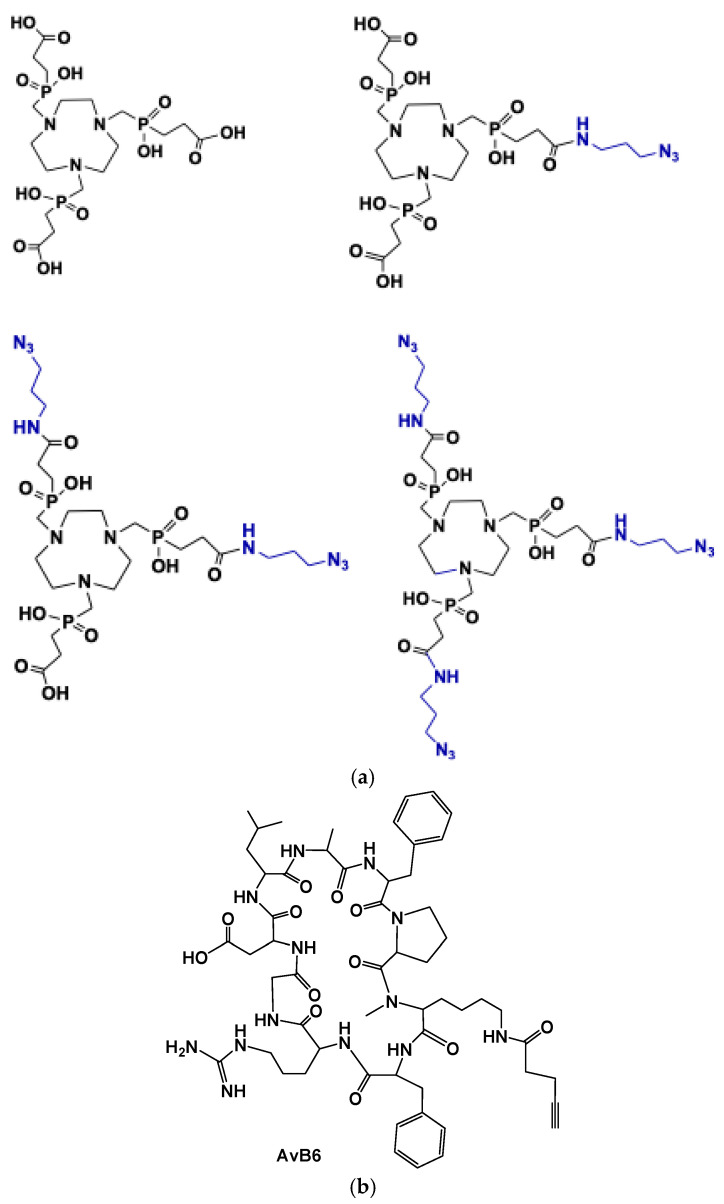
(**a**) TRAP chelators used for the synthesis of integrin AvB6 conjugates by means of click chemistry (Cu-catalized azide-alkyne cycloaddition) with the functionalized AvB6 (**b**).

Over the years, the biological significance of the αvβ6 integrin has driven extensive research into the development of both peptidic and non-peptidic inhibitors specifically targeting this integrin. Among these, several peptide-based ligands have been explored for imaging applications, including the linear peptides A20FMDV2 (NAVPNLRGDLQVLAQKVART), derived from the foot-and-mouth disease virus (FMDV), and H2009.1 (RGDLATLRQL), as well as the cyclic peptide S02 [28]. These ligands have been radiolabeled and successfully employed for in vivo imaging of αvβ6 expression using SPECT and PET techniques [29,30,31,32]. Building on these advances, researchers sought to further refine integrin-targeting ligands by enhancing their metabolic stability and reducing their size while maintaining high affinity. This effort led to the discovery of the cyclic nonapeptide cyclo(FRGDLAFp(NMe)K), a highly potent αvβ6 ligand with a binding affinity of 0.26 nM [33]. Notably, it demonstrated exceptional selectivity against other integrins, with significantly lower affinities for αvβ3, α5β1, αvβ5, and αIIbβ3. Additionally, it exhibited full stability in human plasma for up to three hours. Crucially, functionalization of the lysine side chain did not compromise its activity, making it an ideal scaffold for the development of radiolabeled molecular probes for imaging αvβ6 expression (Figure 1).

**Figure 2:**
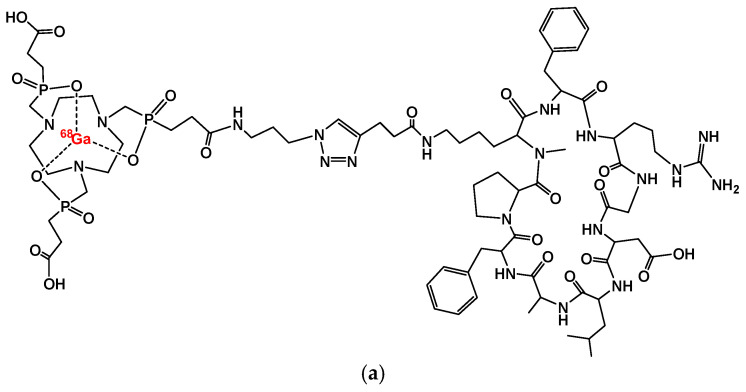

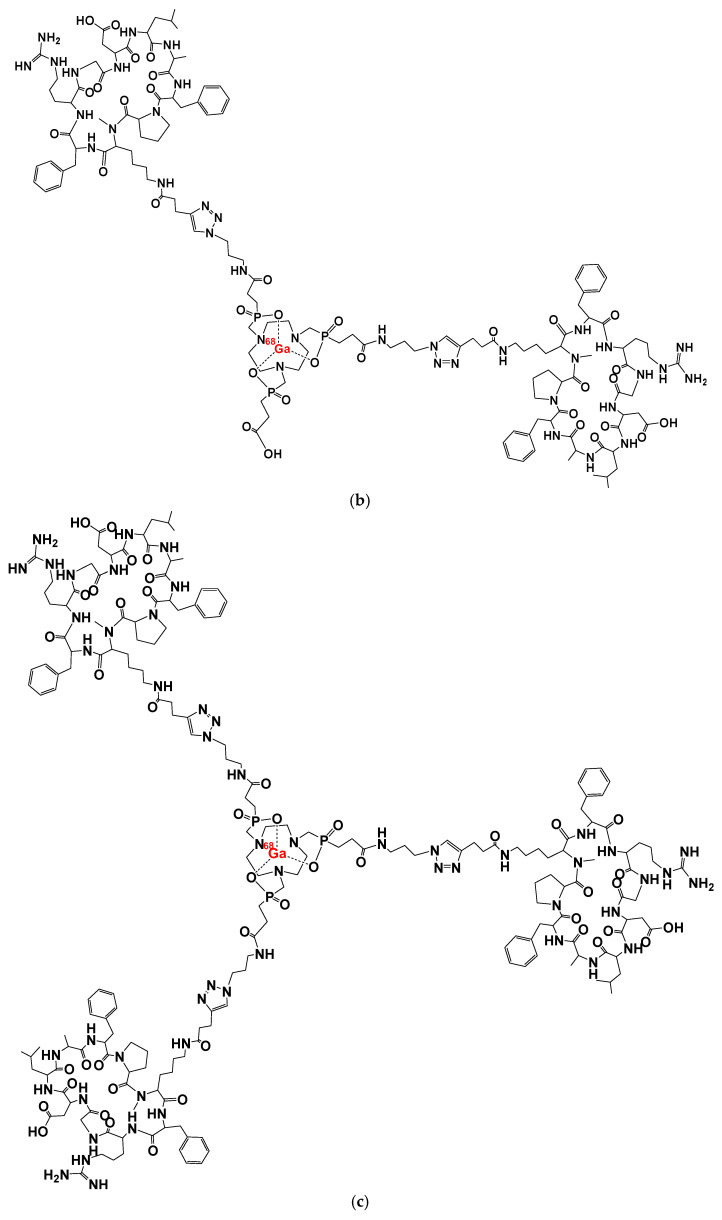
(**a**) [^68^Ga]Ga-Avebehexin; (**b**) the dimer [^68^Ga]Ga-TRAP(AvB6)_2_; (**c**) the timer [^68^Ga]Ga-TRAP(AvB6)_3_ [20].

Notni et al. [34] developed corresponding probes for PET imaging, labeled with ^68^Ga (t_1/2_ = 68 min), which is readily available from ^68^Ge/^68^Ga generators—compact benchtop devices that serve as long-lived regenerative sources of [^68^Ga]Ga^3+^ in dilute HCl. For ^68^Ga radiolabeling, the TRAP (1,4,7-triazacyclononane-1,4,7-tris[methylene(2-carboxyethyl)]phosphinic acid) chelator (Figure 1) was chosen for its high affinity and selectivity for gallium, ensuring efficient and reliable labeling. Additionally, its three conjugation sites allow for easy attachment of reporter molecules or multimerization of targeting vectors, which can be conveniently achieved using click chemistry (Cu-catalyzed azide-alkyne cycloaddition, CuAAC) to modify the probe structure (Figure 2).

Unfortunately, they found that, unlike previous studies, multimerization of the αvβ6-selective peptide c(FRGDLAFp(NMe)K) did not enhance tumor accumulation in PET imaging. While multimers exhibited higher αvβ6 affinity, enhanced by approximately 2-fold for the dimer [^68^Ga]Ga-TRAP(AvB6)_2_ and by approximately 11-fold for the trimer [^68^Ga]Ga-TRAP(AvB6)_3_, compared to the monomeric TRAP-conjugate [^68^Ga]Ga-Avebehexin, they showed inferior pharmacokinetics compared to monomers. In contrast, the [^68^Ga]Ga-Avebehexin demonstrated excellent renal clearance and a low background signal, enabling highly sensitive PET imaging even in tumors with moderate αvβ6 expression (e.g., H2009 lung adenocarcinoma xenografts in mice).

**Figure 3:**
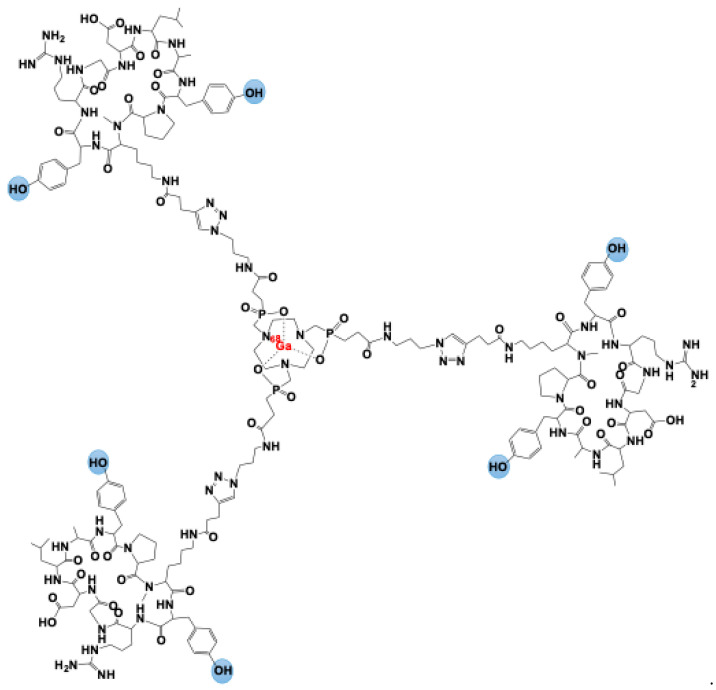
[^68^Ga]Ga-Trivehexin.

The [^68^Ga]Ga-Trivehexin tracer originates from the optimization of the ^68^Ga-labeled TRAP trimer [^68^Ga]Ga-TRAP(AvB6)_3_ [20]. To enhance the hydrophilicity of the conjugates, both phenylalanine residues in c[FRGDLAFp(NMe)K] were substituted with tyrosine (Figure 3). At the atomic level, the structural variations are limited to the presence of two extra oxygen atoms on Tyr2 (sequence: c[YRGDLAYp(NMe)K]), highlighted in blue in Figure 3.

The trimeric conjugate Trivehexin was synthesized using a method similar to that employed for TRAP(AvB6)_3_ and the previously developed αvβ8-integrin tracer [^68^Ga]Ga-Triveoctin [35]. The process involved conjugating the Lys(pentynoic amide) derivative of Tyr2 to the symmetrical TRAP chelator scaffold through a straightforward click chemistry (CuAAC) reaction. This synthesis route facilitated efficient automated ^68^Ga labeling of Trivehexin (5 nmol), performed at pH 2, yielding [^68^Ga]Ga-Trivehexin with a radiochemical efficiency exceeding 95% and a purity of over 99%.

The individual substitution of Phe with Tyr in c[FRGDLAFp(NMe)K] resulted in only a slight decrease in affinity (IC_50_ values of 0.39 pM and 0.40 pM for c[FRGDLAYp(NMe)K] and c[YRGDLAFp(NMe)K], respectively, compared to 0.26 pM for the original peptide). However, the simultaneous replacement of both Phe residues led to a significant drop in affinity and reduced subtype selectivity in the Tyr_2_-alkyne building block compared to its phenylalanine counterpart. Despite this drawback, trimerization effectively compensated for the reduced affinity, yielding an 18-fold increase in αvβ6-integrin binding and a notable improvement in selectivity, consistent with previous findings. Furthermore, [^68^Ga]Ga-Trivehexin demonstrated the anticipated increase in hydrophilicity, with a logD_7.4_ value of −2.1 ± 0.1, which was moderately lower than that of [^68^Ga]Ga-TRAP(AvB6)_3_ (log D_7.4_ = −1.5 ± 0.1) [20].

The evaluation of [^68^Ga]Ga-Trivehexin in cellular and in vivo models provided strong evidence of its specificity and favorable pharmacokinetics. In cellular assays, the tracer demonstrated a specific and blockable uptake in H2009 lung adenocarcinoma cells, which express αvβ6-integrin, while no uptake was observed in MDA-MB-231 breast cancer cells, which lack αvβ6-integrin expression. These results confirmed the tracer’s high selectivity for αvβ6-integrin [20].

When tested in SCID mice bearing H2009 (αvβ6-positive) and MDA-MB-231 (αvβ6-negative) xenografts, dynamic µPET imaging revealed that [^68^Ga]Ga-Trivehexin cleared from the bloodstream significantly faster than [^68^Ga]Ga-TRAP(AvB6)_3_. Notably, [^68^Ga]Ga-Trivehexin exhibited minimal non-specific accumulation in major organs, a clear improvement over [^68^Ga]Ga-TRAP(AvB6)_3_, which showed off-target uptake, particularly in the liver, lungs, and even tumor tissue. This reduced background uptake contributed to better tumor contrast in µPET imaging [20].

The improved biodistribution of [^68^Ga]Ga-Trivehexin led to higher tumor-to-organ ratios, enhancing tumor delineation in µPET images due to the lower background signal. Additionally, PET imaging and biodistribution studies in MDA-MB-231 xenografts further confirmed specificity, as no [^68^Ga]Ga-Trivehexin uptake was detected in these β6-negative tumors. Immunohistochemistry (β6-IHC) verified that MDA-MB-231 xenografts lacked β6-integrin expression, in contrast to H2009 tumors, further validating the selectivity of [^68^Ga]Ga-Trivehexin [20].

To further support translational research, an improved protocol for the radiosynthesis of [^68^Ga]Ga-Trivehexin was developed based on an updated version of the previously described method [20]. This optimization was essential to ensure high yield and high radiochemical purity (RCP) in a clinical setting [36]. Several key modifications were introduced to achieve this goal. First, a variable amount of Trivehexin (30–50 µg) was used to determine the optimal concentration for maximizing efficiency. It was prepared in a sodium acetate buffer (60 mg in 1.5 mL sterile H_2_O, pH = 3.5–4), which provides a stable pH environment crucial for the reaction. Additionally, the ^68^Ge/^68^Ga generator was manually eluted with 5 mL of 0.05 M HCl, ensuring controlled and reproducible gallium-68 extraction. Manual elution allows for precise radionuclide handling, reducing variability and ensuring consistent quality across multiple syntheses.

## 3. [^68^Ga]Ga-Trivehexin: Normal Biodistribution and Dosimetry

Whole-body [^68^Ga]Ga-Trivehexin PET images showed a biodistribution generally characterized by low soft tissue uptake. Apart from renal excretion and partial retention, significant uptake was observed only in the gastric mucosa and the small intestine. Incidental uptake in uterine fibroids with integrin PET imaging was also noted [37,38].

A few feasibility studies investigated the in vivo biodistribution of [^68^Ga]Ga-Trivehexin PET. Thakral et al. [36] performed whole-body PET/CT scans at variable time points (10, 60 and 90 min) post-intravenous (i.v.) injection of 84–185 MBq of 68Ga-Trivehexin to assess its biodistribution and maximum uptake time. They imaged 20 patients with head and neck cancer (HNC) and PDAC and found that [^68^Ga]Ga-Trivehexin PET should ideally be performed at time points >60 min post-injection. Similarly, Rehm et al. [39] investigated the optimal timing for [^68^Ga]Ga-Trivehexin PET/CT. The authors conducted an early dynamic scan in list mode for 45 min post-injection. Notably, the highest target-to-background ratio was observed at 45 min, thus justifying the late scan at 1 h post-injection performed by most authors in the published literature.

A couple of papers explored the dosimetry of [^68^Ga]Ga-Trivehexin PET/CT. With a biological half-life of 10 h (renal excretion) and an assumed urinary bladder residence of 0.07 h, dosimetry calculations using OLINDA V1.1 yielded a moderate effective dose (ICRP 60) of 3.36 × 10^−2^ mSv/MBq, or 4.7 mSv for an injection of 148 MBq (4 mCi) [^68^Ga]Ga-Trivehexin [20]. Further dosimetry analysis by Wang et al. [40] revealed a total body effective dose of 1.67 × 10^−2^ mSv/MBq, with the highest effective dose in the kidneys (2.26 × 10^−1^ mSv/MBq), followed by the urinary bladder wall (8.24 × 10^−2^ mSv/MBq) and adrenals (3.22 × 10^−2^ mSv/MBq).

## 4. [^68^Ga]Ga-Trivehexin: Preliminary Clinical Experiences

To date, [^68^Ga]Ga-Trivehexin PET has been performed only in a very limited number of clinical experiences. We performed a literature search in PubMed, Scopus and Web of Science. The last research was run on 31 March 2025, in which the following keywords were combined: “trivehexin and PET”. Overall, 16 articles have been published, involving a total of 124 patients imaged with [^68^Ga]Ga-Trivehexin PET. Among those, most were case reports (n. 11; 68.7%). Most of the studies (n. 15; 93.7%) involved oncologic patients (n. of patients: 111; 89.5%), but three studies (18.7%) also reported on [^68^Ga]Ga-Trivehexin PET in non-oncological conditions (n. of patients: 15; 12.1%). Of note, two case reports have described [^68^Ga]Ga-Trivehexin PET in patients affected by both oncological and non-oncological diseases overexpressing αvβ6-integrin. Among oncologic patients, the main clinical indications for the scan were (a) pancreatic ductal adenocarcinoma—PDAC (n. of patients: 56); and (b) head and neck squamous cell carcinoma—HNSCC (n. of patients: 23). Clinical experiences with [^68^Ga]Ga-Trivehexin PET in oncological and non-oncological patients are listed in Table 1.

### 4.1. Oncological Diseases

The first clinical experience with [^68^Ga]Ga-Trivehexin PET/Computed Tomography (CT) has been reported by Quigley et al. [41] in a patient affected by PDAC. Intense focal uptake could be seen at the primary tumor at the pancreatic head. The same authors [20] imaged four patients—one healthy volunteer for the assessment of the radiotracer’s biodistribution and dosimetry calculations and three oncological patients—with [^68^Ga]Ga-Trivehexin PET/CT. In the healthy volunteer, the scan was performed at three different time points (13, 44 and 97 min post injection). The biodistribution of [^68^Ga]Ga-Trivehexin PET/CT demonstrated high radiotracer concentration in the kidneys—referable to the known renal excretion of the radiotracer—but low uptake in other soft tissues. Of note, the low diffuse uptake detectable in the gastric mucosa and in the small intestine at the early scan decreased progressively in images acquired after 44 min and was nearly absent in images acquired 97 min post injection. Thus, the optimal time point for a scan with [^68^Ga]Ga-Trivehexin PET/CT should be at least 60 min post injection. Among oncological patients (one HNSCC, one salivary duct carcinoma and one PDAC), [^68^Ga]Ga-Trivehexin PET/CT detected the same sites of oncological disease identified at [^18^F]FDG PET/CT in the patients affected by HNSCC and salivary duct carcinoma. Of note, [^68^Ga]Ga-Trivehexin PET/CT showed lower inflammatory uptake surrounding the tracheostomy of the HNSCC patient and did not show mediastinal reactive lymph nodes in the patient with salivary duct carcinoma. In the PDAC patient, [^68^Ga]Ga-Trivehexin PET/CT detected more liver metastases than CT. An analogous experience has been reported by Das et al. [42]. The authors imaged 5 patients without a history of cancer and 32 oncological patients (20 HNSCC, 9 PDAC, 2 other histotypes of pancreatic cancers and 1 colon cancer infiltrating the pancreatic head) with [^68^Ga]Ga-Trivehexin PET/CT. In the oncologic group, the authors compared the scan results with those of standard of care [^18^F]FDG PET/CT and investigated a correlation between tumor uptake at [^68^Ga]Ga-Trivehexin PET/CT and αvβ6-integrin expression at immunohistochemistry (IHC). Of note, biopsies were performed in the primary tumor and/or metastases, thus representing an ideal standard of reference. Among nine PDAC imaged, eight showed focal [^68^Ga]Ga-Trivehexin uptake, and two of those were negative at [^18^F]FDG PET/CT. Conversely, one case of PDAC resulted in a negative [^68^Ga]Ga-Trivehexin PET/CT, and another case with higher uptake at [^18^F]FDG than at [^68^Ga]Ga-Trivehexin PET/CT was found. In both cases, histology demonstrated a poorly differentiated PDAC. Among HNSCC, [^68^Ga]Ga-Trivehexin PET/CT showed intense uptake in all cases except one (tracheal carcinoma). Conversely, in two patients with HNSCC, a suspected recurrence detected at [^18^F]FDG PET/CT was ruled out by [^68^Ga]Ga-Trivehexin PET/CT and later confirmed as inflammatory changes at biopsy. Overall, [^68^Ga]Ga-Trivehexin PET/CT demonstrated a higher target-to-background ratio than [^18^F]FDG PET/CT in both types of cancer, with a sensitivity and specificity of 92.5% and 100%, respectively. Among biopsy-confirmed cancer lesions, a positive correlation between SUVmax at [^68^Ga]Ga-Trivehexin PET/CT and αvβ6-integrin expression at IHC was found (*p* < 0.0001). Noteworthy, the [^68^Ga]Ga-Trivehexin uptake was often more intense at the periphery of the cancer lesion, in analogy with αvβ6-integrin expression.

In the study with the larger cohort currently published in the literature, Rehm et al. [39] investigated 44 PDAC patients with [^68^Ga]Ga-Trivehexin PET/CT. The authors had a double aim. The first was to identify the best timing for [^68^Ga]Ga-Trivehexin PET/CT, as discussed in the previous section of this article. The second aim was to evaluate the accuracy of [^68^Ga]Ga-Trivehexin PET/CT to stage PDAC patients, both locally and at a distance. [^68^Ga]Ga-Trivehexin PET/CT identified the primary tumor in 40 out of 44 cases. Furthermore, 21 lymph node metastases, 39 liver metastases, 17 peritoneal metastases and 14 metastases in other sites were identified. The highest uptake intensity—expressed in terms of maximum standardized uptake value (SUVmax)—was identified in correspondence with the primary pancreatic tumor with an average SUVmax = 12.6 (5.1–30.8).

Furthermore, several case reports have been published. In the work by Rehm and colleagues [43], a patient affected by both HNSCC and PDAC was imaged with [^68^Ga]Ga-Trivehexin PET/CT. Along with both primary tumors, the scan detected a brain metastasis in the cerebellopontine angle with high [^68^Ga]Ga-Trivehexin uptake. Indeed, the identification of that finding would have been very difficult at [^18^F]FDG PET/CT due to the high physiological tracer uptake in the gray matter. Conversely, the very low background at [^68^Ga]Ga-Trivehexin PET/CT enabled an easy detection of the brain metastasis. Other case reports highlighting the potential of [^68^Ga]Ga-Trivehexin PET/CT in patients with non-small cell lung cancer, ovarian carcinoma, breast cancer, bronchial mucoepidermoid carcinoma, solid pseudopapillary neoplasm of the pancreas and papillary thyroid carcinoma have been published [44,45,46,47,48,49,50,51]. Many of these case reports emphasize the ability of [^68^Ga]Ga-Trivehexin PET/CT to exclude cancer persistence in inflammatory findings positive at [^18^F]FDG PET/CT.

### 4.2. Non-Oncological Diseases

Although most clinical experiences with [^68^Ga]Ga-Trivehexin PET/CT have been performed in oncological patients, a few preliminary scans have been performed in non-oncological conditions too. Wu and colleagues [51] showed that idiopathic pulmonary fibrosis (IPF)—a progressive disease with poor prognosis, sustained by the development of fibrosis due to an excessive uncontrolled deposition of ECM in the lungs—is associated with diffuse, intense [^68^Ga]Ga-Trivehexin uptake [52]. Similar results have been recently reported by Kuyumcu et al. [53]. This finding paves the way for further experiences aiming to define the possible role of [^68^Ga]Ga-Trivehexin PET/CT to diagnose IPF. Moreover, [^68^Ga]Ga-Trivehexin PET/CT could potentially enable the assessment of the dynamic process of fibrosis, allowing visualization of the response to new treatments like Nintedanib [54].

**Figure 4:**
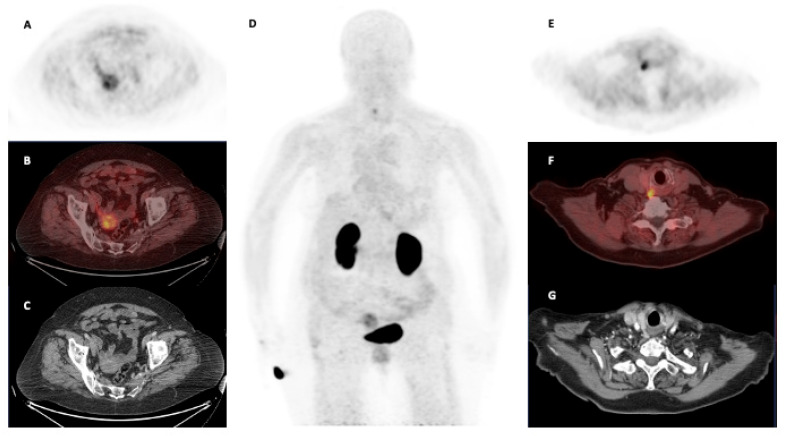
Normal biodistribution of [^68^Ga]Ga-Trivehexin PET/CT in a patient with primary hyperparathyroidism performed at the Department of Nuclear Medicine, Hacettepe University, Ankara, Turkey. PET/CT was acquired 60 min p.i. of 160 MBq of [^68^Ga]Ga-Trivehexin and characterized by low soft tissue uptake ((**A**,**E**): emissive trans-axial PET images; (**B**,**F**): trans-axial fused PET/CT images; (**C**,**G**): trans-axial CT images; (**D**): maximum intensity projection—MIP). Radiotracer uptake was seen in the kidneys and bladder due to renal excretion. Incidental uptake in a uterine fibroid was also noted (**A**–**C**). A focal radiotracer uptake in a parathyroid adenoma was detected.

Finally, in analogy to what has already happened with most radiotracers available, the progressive clinical use determines the identification of incidental findings that may increase the initial applications of the imaging method [55]. Following the incidental detection of a parathyroid adenoma with focal uptake at [^68^Ga]Ga-Trivehexin PET/CT, Kuyumcu and colleagues [56] scanned 13 patients with primary hyperthyroidism (PHP). [^68^Ga]Ga-Trivehexin PET/CT was performed within 3–7 days from [^99m^Tc]Tc-MIBI SPECT/CT. Moreover, three patients were also scanned with [^18^F]F-choline PET/CT, which has been demonstrated to identify parathyroid adenomas in a large proportion of patients with negative [^99m^Tc]Tc-MIBI SPECT/CT scans [57] (Figure 4). [^68^Ga]Ga-Trivehexin PET/CT was positive in 11 out of 13 PHP patients, detecting a total of 16 parathyroid adenomas, 7 more than [^99m^Tc]Tc-MIBI SPECT/CT. Overall, the detection rate of [^68^Ga]Ga-Trivehexin PET/CT was 94.1%. Furthermore, [^68^Ga]Ga-Trivehexin PET/CT yielded positive scans in 2 patients with negative [^18^F]F-choline PET/CT.

**Table 1:** Clinical experiences with [^68^Ga]Ga-Trivehexin PET in oncological and non-oncological patients.

Author	Country	Year	Clinical Setting	Disease	N. of Patients	Injected Activity (MBq)	Time of Scan (mins p.i.)	Main Findings
Quigley et al. [41]	Germany	2021	Oncological	PDAC	1	87	70	Focal radiotracer uptake at the PDAC of the pancreatic head.
Quigley et al. [20]	Germany	2022	Oncological	HNSCC, salivary duct carcinoma, PDAC	4	172–142–135–105	61–120	[^68^Ga]Ga-Trivehexin PET/CT detected the same oncological lesions visible at [^18^F]FDG PET/CT but with lower inflammatory findings in HNSCC and salivary duct carcinoma. In PDAC, [^68^Ga]Ga-Trivehexin PET/CT revealed more liver metastases than CT.
Thakral et al. [36]	India	2023	Oncological	HNC, PDAC	20	110 (84–185)	10, 60, 90	The scan performed 60 min post injection demonstrated the higher radiotracer uptake.
Rehm et al. [43]	Germany	2024	Oncological	HNSCC, PDAC	1	146	60	[^68^Ga]Ga-Trivehexin PET/CT detected a brain metastasis from HNSCC.
Das et al. [42]	India—Germany	2023	Oncological	HNC, PDAC	32	1.85–2.22 per kg	60	[^68^Ga]Ga-Trivehexin PET/CT showed a sensitivity and specificity of 92.5% and 100%, respectively. Uptake intensity showed a positive correlation with αvβ6-integrin expression at IHC.
Marafi et al. [44]	Kuwait	2024	Oncological	NSCLC	1	–	–	[^68^Ga]Ga-Trivehexin PET/CT detected brain metastases from NSCLC.
Singh et al. [45]	India	2024	Oncological	Ovarian carcinoma	1	–	–	[^68^Ga]Ga-Trivehexin PET/CT may be useful to define disease extension in metastatic ovarian carcinoma.
Wu et al. [47]	China	2024	Oncological	Bronchial mucoepidermoid carcinoma	1	–	–	[^68^Ga]Ga-Trivehexin PET demonstrated higher TBR than [^18^F]FDG PET/CT in a patient with bronchial mucoepidermoid carcinoma (10.1 vs. 2.5, respectively).
Novruzov et al. [49]	Azerbaijan	2024	Oncological	SPPNP	1	–	–	[^68^Ga]Ga-Trivehexin PET/CT was negative in a patient with SPPNP with high uptake at [^18^F]FDG and [^68^Ga]Ga-FAPI PET/CT.
Rehm et al. [39]	Germany	2024	Oncological	PDAC	44	139 (84–160)	Early dynamic: 45 min acquisition in list mode; Late scan: 62 (55–85)	[^68^Ga]Ga-Trivehexin PET/CT demonstrated high accuracy for the detection of αvβ6-integrin expression in pancreatic cancer and metastases (lymph node, liver, peritoneal and other sites). The dynamic acquisition showed that the latest images (acquired 45 min p.i) had the higher TBR.
Kuyumcu et al. [56]	Turkey	2024	Non-oncological	PHP	13	185	Early scan: 30 Late scan: 50–90	In comparison to [^99m^Tc]Tc-MIBI scintigraphy-SPECT/CT, [^68^Ga]Ga-Trivehexin PET/CT identified 7 additional parathyroid lesions. [^68^Ga]Ga-Trivehexin PET/CT identified hyperfunctioning parathyroids in 2 out of 3 PHP patients negative at [^18^F]fluorocholine PET/CT.
Singhal et al. [46]	India	2025	Oncological	DTC	1	–	–	[^68^Ga]Ga-Trivehexin PET/CT was more accurate than [^18^F]FDG PET/CT to define disease extension in a patient with papillary thyroid carcinoma.
Kömek et al. [48]	Turkey	2025	Oncological	BC	1	–	–	[^68^Ga]Ga-Trivehexin PET/CT detected the primary BC, left internal mammary and axillary lymph node metastases with a higher tracer uptake than [^18^F]FDG PET/CT. Moreover, ruled out disease involvement in mediastinal [^18^F]FDG-avid lymph nodes, confirmed reactive at cytology.
Emerson et al. [50]	India	2025	Oncological	HNSCC	1	–	–	[^68^Ga]Ga-Trivehexin PET/CT detected HNSCC of the oral cavity.
Wu et al. [51]	China	2025	Oncological/Non-oncological	NSCLC, IPF	1	–	–	[^68^Ga]Ga-Trivehexin PET/CT showed intense uptake in correspondence to a mucinous lung adenocarcinoma (with only faint uptake at [^18^F]FDG PET/CT) and IPF.
Kuyumcu et al. [53]	Turkey	2025	Oncological/Non-oncological	NSCLC, IPF	1	–	–	A mucinous lung cancer demonstrated higher uptake at [^68^Ga]Ga-Trivehexin than at [^18^F]FDG PET/CT. [^68^Ga]Ga-Trivehexin demonstrated diffuse intense uptake in IPF.

BC = breast cancer; CT = Computed Tomography; DTC = differentiated thyroid carcinoma; FAPI = fibroblast activating protein inhibitor; HNC = head and neck cancer; HNSCC = head and neck squamous cell carcinoma; IHC = immunohistochemistry; IPF = idiopathic pulmonary fibrosis; NSCLC = non-small cell lung cancer; PDAC = pancreatic ductal adenocarcinoma; PET = positron emission tomography; p.i. = post injection; PHP = primary hyperparathyroidism; SPECT/CT = Single Photon Emission Tomography/Computed Tomography; SPPNP = solid pseudopapillary neoplasm of the pancreas; TBR = target-to-background ratio.

## 5. Discussion

[^68^Ga]Ga-Trivehexin represents a significant advancement in the field of radiochemistry applied to molecular diagnostics for PET imaging of tumors expressing αvβ6 integrin. This radiopharmaceutical is based on an optimized multimeric system, [⁶⁸Ga]Ga-TRAP(AvB6)_3_, [20], designed to enhance pharmacokinetic properties, biodistribution, and binding specificity compared to previous monomeric tracers [34,35]. To enhance the hydrophilicity of the conjugates, both phenylalanine residues in c[FRGDLAFp(NMe)K] were substituted with tyrosine. This substitution effectively enhances the hydrophilicity of the conjugates while maintaining the overall structural integrity, with only minor atomic-level modifications due to the additional oxygen atoms in the tyrosine residues.

The synthesis protocol for [^68^Ga]Ga-Trivehexin was developed and optimized from previously described methods, with targeted modifications to improve yield and radiochemical purity. Crucial parameters for achieving good labeling yield are the pH of the reaction, the amount of peptide, and the reaction time. Once again, the optimal pH and labeling temperature proved to be the same as those used for the preparation of [^68^Ga]Ga-PSMA and [^68^Ga]Ga-DOTANOC (pH 3.5–4 and T = 90 °C) [36]. The final purification based on a C8 cartridge ensures high radiochemical purity (>95%), essential for safe and effective clinical use.

The incorporation of multimeric Trivehexin has significantly improved binding properties compared to the monomeric [^68^Ga]Ga-Avebehexin, as increased affinity for αvβ6 integrin, with up to an 18-fold enhancement compared to monomers, greater selectivity due to the avidity effect generated by the trimeric structure, and improved pharmacokinetics, characterized by faster accumulation in target tissues and efficient clearance from non-specific compartments [20,41].

Overall, these findings highlight [^68^Ga]Ga-Trivehexin as a highly selective and stable PET tracer with rapid clearance, low non-specific organ uptake, and improved imaging contrast, making it a promising tool for αvβ6-integrin imaging in tumors.

Considering clinical applications of [^68^Ga]Ga-Trivehexin PET/CT, the best timing for the scan appears to be at 60 min p.i. Too early acquisition may be impaired by non-specific tracer distribution (i.e., in the gastric mucosa and in the small intestine) [20]. Conversely, late acquisitions may show poor image quality due to the short half-life of the radionuclide [^68^Ga]Ga (68 min).

Currently, among oncological patients, [^68^Ga]Ga-Trivehexin PET/CT has been mostly investigated in HNC and PDAC [36,39,42]. In both conditions, [^68^Ga]Ga-Trivehexin PET/CT has demonstrated high detection rates in the preliminary data available in the literature. In HNC, the main advantage of [^68^Ga]Ga-Trivehexin PET/CT over [^18^F]FDG PET/CT might be the differential diagnosis between oncological and inflammatory findings [36,42]. [^18^F]FDG uptake is sustained by increased glucose metabolism and thus is unspecific. Conversely, [^68^Ga]Ga-Trivehexin directly targets αvβ6-integrin, which is overexpressed in epithelial cancers but negative in inflammatory lymph nodes or reactive tissues, which are frequently associated with HNC [58]. Therefore, [^68^Ga]Ga-Trivehexin PET/CT could become a useful tool for the differential diagnosis between inflammatory and oncological findings that are undetermined at [^18^F]FDG PET/CT. In PDAC patients, [^68^Ga]Ga-Trivehexin PET/CT might become even more relevant. [^18^F]FDG PET/CT is often negative or shows only faint uptake in PDAC and is not routinely used [59,60,61,62]. In PDAC patients, [^68^Ga]Ga-Trivehexin PET/CT might play a complementary role to conventional radiologic imaging (i.e., CT or magnetic resonance imaging), in particular for the detection of distant metastases [39].

Furthermore, [^68^Ga]Ga-Trivehexin PET/CT may carve out a role in non-oncological conditions as well. IPF is associated with increased deposition of ECM in the lungs and showed diffuse high uptake at [^68^Ga]Ga-Trivehexin PET/CT in a couple of case reports [51,53]. Indeed, further experiences are needed to confirm that IPF is associated with high [^68^Ga]Ga-Trivehexin uptake. However, [^68^Ga]Ga-Trivehexin PET/CT may become a pivotal imaging in this severe progressive disease, enabling the definition of the real extent of the condition and the deposition rate of ECM in the lungs during therapies, which are very difficult tasks to address with currently available imaging modalities [63,64]. Furthermore, the incidental identification of increased [^68^Ga]Ga-Trivehexin uptake in a parathyroid adenoma led to a preliminary research article by Kuyumcu et al. [56]. [^68^Ga]Ga-Trivehexin PET/CT detected more parathyroid adenomas than [^99m^Tc]Tc-MIBI SPECT/CT and [^18^F]F-choline PET/CT (Figure 4). However, [^18^F]F-choline PET/CT was performed only in a few patients. Therefore, a head-to-head trial comparing the detection rates of [^18^F]F-choline and [^68^Ga]Ga-Trivehexin PET/CT is desirable to define if [^68^Ga]Ga-Trivehexin PET/CT might have a role among imaging procedures of patients with PHP.

## 6. Conclusions

[^68^Ga]Ga-Trivehexin is an emerging PET probe for in vivo visualization of αvβ6-integrin, which is highly overexpressed in several epithelial cancers correlated with the TGF-β pathway. In both HNC and PDAC, [^68^Ga]Ga-Trivehexin PET/CT is promising and deserves to be further investigated to define its real potentialities and indications. Moreover, non-oncological diseases like IPF and PHP showed increased uptake at [^68^Ga]Ga-Trivehexin PET/CT in preliminary clinical reports. Further literature evidence is needed to investigate the accuracy of [^68^Ga]Ga-Trivehexin PET/CT in these diseases.

## Data Availability

Not applicable.

## References

[1] Lawal I.O., Abubakar S.O., Ndlovu H., Mokoala K.M.G., More S.S., Sathekge M.M. (2024). Advances in Radioligand Theranostics in Oncology. Mol. Diagn. Ther..

[2] Vaz S.C., Woll J.P.P., Cardoso F., Groheux D., Cook G.J.R., Ulaner G.A., Jacene H., Rubio I.T., Schoones J.W., Peeters M.-J.V. (2024). Joint EANM-SNMMI Guideline on the Role of 2-[18F]FDG PET/CT in No Special Type Breast Cancer: (Endorsed by the ACR, ESSO, ESTRO, EUSOBI/ESR, and EUSOMA). Eur. J. Nucl. Med. Mol. Imaging.

[3] Urso L., Panareo S., Castello A., Ambrosio M.R., Zatelli M.C., Caracciolo M., Tonini E., Valpiani G., Boschi A., Uccelli L. (2022). Glucose Metabolism Modification Induced by Radioligand Therapy with [177Lu]Lu/[90Y]Y-DOTATOC in Advanced Neuroendocrine Neoplasms: A Prospective Pilot Study within FENET-2016 Trial. Pharmaceutics.

[4] Urso L., Quartuccio N., Caracciolo M., Evangelista L., Schirone A., Frassoldati A., Arnone G., Panareo S., Bartolomei M. (2022). Impact on the Long-Term Prognosis of FDG PET/CT in Luminal-A and Luminal-B Breast Cancer. Nucl. Med. Commun..

[5] Castello A., Rossi S., Lopci E. (2020). 18F-FDG PET/CT in Restaging and Evaluation of Response to Therapy in Lung Cancer: State of the Art. Curr. Radiopharm..

[6] Casali M., Lauri C., Altini C., Bertagna F., Cassarino G., Cistaro A., Erba A.P., Ferrari C., Mainolfi C.G., Palucci A. (2021). State of the Art of 18F-FDG PET/CT Application in Inflammation and Infection: A Guide for Image Acquisition and Interpretation. Clin. Transl. Imaging.

[7] Evangelista L., Zattoni F., Burei M., Bertin D., Borsatti E., Baresic T., Farsad M., Trenti E., Bartolomei M., Panareo S. (2024). A Prospective Randomized Multicenter Study on the Impact of [18F]F-Choline PET/CT Versus Conventional Imaging for Staging Intermediate- to High-Risk Prostate Cancer. J. Nucl. Med..

[8] Chondrogiannis S., Marzola M.C., Rubello D. (2014). ^18^F-DOPA PET/Computed Tomography Imaging. PET Clin..

[9] Dondi F., Gazzilli M., Viganò G.L., Pisani A.R., Ferrari C., Rubini G., Bertagna F. (2024). The Role of 11C-Methionine PET Imaging for the Evaluation of Lymphomas: A Systematic Review. Hematol. Rep..

[10] Calais J., Ceci F., Eiber M., Hope T.A., Hofman M.S., Rischpler C., Bach-Gansmo T., Nanni C., Savir-Baruch B., Elashoff D. (2019). 18F-Fluciclovine PET-CT and 68Ga-PSMA-11 PET-CT in Patients with Early Biochemical Recurrence after Prostatectomy: A Prospective, Single-Centre, Single-Arm, Comparative Imaging Trial. Lancet Oncol..

[11] Hofman M.S., Lau W.F.E., Hicks R.J. (2015). Somatostatin Receptor Imaging with 68Ga DOTATATE PET/CT: Clinical Utility, Normal Patterns, Pearls, and Pitfalls in Interpretation. Radiographics.

[12] Hofman M.S., Lawrentschuk N., Francis R.J., Tang C., Vela I., Thomas P., Rutherford N., Martin J.M., Frydenberg M., Shakher R. (2020). Prostate-Specific Membrane Antigen PET-CT in Patients with High-Risk Prostate Cancer before Curative-Intent Surgery or Radiotherapy (proPSMA): A Prospective, Randomised, Multicentre Study. Lancet.

[13] Filippi L., Urso L., Bianconi F., Palumbo B., Marzola M.C., Evangelista L., Schillaci O. (2023). Radiomics and Theranostics with Molecular and Metabolic Probes in Prostate Cancer: Toward a Personalized Approach. Expert Rev. Mol. Diagn..

[14] Sartor O., De Bono J., Chi K.N., Fizazi K., Herrmann K., Rahbar K., Tagawa S.T., Nordquist L.T., Vaishampayan N., El-Haddad G. (2021). Lutetium-177–PSMA-617 for Metastatic Castration-Resistant Prostate Cancer. N. Engl. J. Med..

[15] Duan X., Xia L., Zhang Z., Ren Y., Pomper M.G., Rowe S.P., Li X., Li N., Zhang N., Zhu H. (2023). First-in-Human Study of the Radioligand 68Ga-N188 Targeting Nectin-4 for PET/CT Imaging of Advanced Urothelial Carcinoma. Clin. Cancer Res..

[16] Lindner T., Giesel F.L., Kratochwil C., Serfling S.E. (2021). Radioligands Targeting Fibroblast Activation Protein (FAP). Cancers.

[17] Filippi L., Urso L., Schillaci O., Evangelista L. (2023). [18F]-FDHT PET for the Imaging of Androgen Receptor in Prostate and Breast Cancer: A Systematic Review. Diagnostics.

[18] Kimura R.H., Iagaru A., Guo H.H. (2023). Mini Review of First-in-Human Integrin Avβ6 PET Tracers. Front. Nucl. Med..

[19] Hamidi H., Ivaska J. (2018). Every Step of the Way: Integrins in Cancer Progression and Metastasis. Nat. Rev. Cancer.

[20] Quigley N.G., Steiger K., Hoberück S., Czech N., Zierke M.A., Kossatz S., Pretze M., Richter F., Weichert W., Pox C. (2022). PET/CT Imaging of Head-and-Neck and Pancreatic Cancer in Humans by Targeting the “Cancer Integrin” Avβ6 with Ga-68-Trivehexin. Eur. J. Nucl. Med. Mol. Imaging.

[21] Pierschbacher M.D., Ruoslahti E. (1984). Cell Attachment Activity of Fibronectin Can Be Duplicated by Small Synthetic Fragments of the Molecule. Nature.

[22] Steiger K., Quigley N.G., Groll T., Richter F., Zierke M.A., Beer A.J., Weichert W., Schwaiger M., Kossatz S., Notni J. (2021). There Is a World beyond Avβ3-Integrin: Multimeric Ligands for Imaging of the Integrin Subtypes Avβ6, Avβ8, Avβ3, and A5β1 by Positron Emission Tomography. EJNMMI Res..

[23] Notni J. (2022). RGD Forever!-Past, Present, and Future of a 3-Letter-Code in Radiopharmacy and Life Sciences. Pharmaceuticals.

[24] Schottelius M., Laufer B., Kessler H., Wester H.-J. (2009). Ligands for Mapping α_v_ β_3_ -Integrin Expression in Vivo. Acc. Chem. Res..

[25] Liu S., Chen S., Zeng J. (2017). TGF-β Signaling: A Complex Role in Tumorigenesis (Review). Mol. Med. Rep..

[26] Niu J., Li Z. (2017). The Roles of Integrin Avβ6 in Cancer. Cancer Lett..

[27] Horan G.S., Wood S., Ona V., Li D.J., Lukashev M.E., Weinreb P.H., Simon K.J., Hahm K., Allaire N.E., Rinaldi N.J. (2008). Partial Inhibition of Integrin Alpha(v)Beta6 Prevents Pulmonary Fibrosis without Exacerbating Inflammation. Am. J. Respir. Crit. Care Med..

[28] Li S., McGuire M.J., Lin M., Liu Y.-H., Oyama T., Sun X., Brown K.C. (2009). Synthesis and Characterization of a High-Affinity {alpha}v{beta}6-Specific Ligand for in Vitro and in Vivo Applications. Mol. Cancer Ther..

[29] Hausner S.H., Abbey C.K., Bold R.J., Gagnon M.K., Marik J., Marshall J.F., Stanecki C.E., Sutcliffe J.L. (2009). Targeted In Vivo Imaging of Integrin Avβ6 with an Improved Radiotracer and Its Relevance in a Pancreatic Tumor Model. Cancer Res..

[30] Zhu X., Li J., Hong Y., Kimura R.H., Ma X., Liu H., Qin C., Hu X., Hayes T.R., Benny P. (2014). ^99m^ Tc-Labeled Cystine Knot Peptide Targeting Integrin α_v_ β_6_ for Tumor SPECT Imaging. Mol. Pharm..

[31] Liu Z., Liu H., Ma T., Sun X., Shi J., Jia B., Sun Y., Zhan J., Zhang H., Zhu Z. (2014). Integrin α_v_ β_6_ –Targeted SPECT Imaging for Pancreatic Cancer Detection. J. Nucl. Med..

[32] John A.E., Luckett J.C., Tatler A.L., Awais R.O., Desai A., Habgood A., Ludbrook S., Blanchard A.D., Perkins A.C., Jenkins R.G. (2013). Preclinical SPECT/CT Imaging of Avβ6 Integrins for Molecular Stratification of Idiopathic Pulmonary Fibrosis. J. Nucl. Med..

[33] Maltsev O.V., Marelli U.K., Kapp T.G., Di Leva F.S., Di Maro S., Nieberler M., Reuning U., Schwaiger M., Novellino E., Marinelli L. (2016). Stable Peptides Instead of Stapled Peptides: Highly Potent αvβ6-Selective Integrin Ligands. Angew. Chem. Int. Ed..

[34] Notni J., Reich D., Maltsev O.V., Kapp T.G., Steiger K., Hoffmann F., Esposito I., Weichert W., Kessler H., Wester H.-J. (2017). In Vivo PET Imaging of the Cancer Integrin Avβ6 Using^68^ Ga-Labeled Cyclic RGD Nonapeptides. J. Nucl. Med..

[35] Quigley N.G., Steiger K., Richter F., Weichert W., Hoberück S., Kotzerke J., Notni J. (2020). Tracking a TGF-β Activator in Vivo: Sensitive PET Imaging of Avβ8-Integrin with the Ga-68-Labeled Cyclic RGD Octapeptide Trimer Ga-68-Triveoctin. EJNMMI Res..

[36] Thakral P., Das S.S., Dhiman S., Manda D., Virupakshappa C.B., Malik D., Sen I. (2023). Validation of In-House Kit-Like Synthesis of^68^ Ga-Trivehexin and Its Biodistribution for Targeting the Integrin Avβ6 Expressing Tumors. Cancer Biother. Radiopharm..

[37] Solanki R., Mittal B.R., Kumar R., Singh H., Sharma A. (2024). Incidental Detection of 68 Ga-DOTA-RGD-2 Uptake in Uterine Fibroid. Clin. Nucl. Med..

[38] Ierardi A.M., Carnevale A., Pellegrino F., Stefano G.D., Bonelli C., Renzulli M., Giganti M., Carrafiello G. (2021). Uterine Myomas: Extravascular Treatment. Semin. Ultrasound CT MRI.

[39] Rehm J., Winzer R., Pretze M., Müller J., Notni J., Hempel S., Distler M., Folprecht G., Kotzerke J. (2024). Avβ6-Integrin Targeted PET/CT Imaging in Pancreatic Cancer Patients Using 68Ga-Trivehexin. Front. Nucl. Med..

[40] Wang B., Jiang Y., Zhu J., Wu H., Wu J., Li L., Huang J., Xiao Z., He Y. (2024). Fully-Automated Production of [68Ga]Ga-Trivehexin for Clinical Application and Its Biodistribution in Healthy Volunteers. Front. Oncol..

[41] Quigley N.G., Czech N., Sendt W., Notni J. (2021). PET/CT Imaging of Pancreatic Carcinoma Targeting the “Cancer Integrin” Avβ6. Eur. J. Nucl. Med. Mol. Imaging.

[42] Das S.S., Ahlawat S., Thakral P., Malik D., Simecek J., Cb V., Koley M., Gupta J., Sen I. (2024). Potential Efficacy of 68Ga-Trivehexin PET/CT and Immunohistochemical Validation of Avβ6 Integrin Expression in Patients With Head and Neck Squamous Cell Carcinoma and Pancreatic Ductal Adenocarcinoma. Clin. Nucl. Med..

[43] Rehm J., Winzer R., Notni J., Hempel S., Distler M., Folprecht G., Kotzerke J. (2024). Concomitant Metastatic Head-and-Neck Cancer and Pancreatic Cancer Assessed by Avβ6-Integrin PET/CT Using 68Ga-Trivehexin: Incidental Detection of a Brain Metastasis. Eur. J. Nucl. Med. Mol. Imaging.

[44] Marafi F., Esmail A.A., Alfeeli M.A., Sadeq A. (2024). 68Ga-Trivehexin PET/CT in Metastatic Non–Small Cell Lung Cancer to the Brain. Clin. Nucl. Med..

[45] Singh P., Agrawal K., Emerson R., Baranwal A., Patro P.S.S., Parida G.K. (2024). “Cancer Integrin” Avβ6 Imaging With 68Ga-Trivehexin PET/CT in Assessment of Ovarian Carcinoma. Clin. Nucl. Med..

[46] Singhal T., Agrawal K., Mandal S., Parida G.K. (2025). Cancer-Specific Integrin Imaging With 68Ga-Trivehexin: A Potential Imaging for Accurate Staging of Thyroid Malignancy. Clin. Nucl. Med..

[47] Wu H., Li L., Xiao Z., Li C., He Y. (2025). Avβ6-Integrin Targeted [68Ga]Ga-Trivehexin PET/CT Imaging of a Rare Bronchial Mucoepidermoid Carcinoma. Eur. J. Nucl. Med. Mol. Imaging.

[48] Kömek H., Güzel Y., Kaplan İ., Yilmaz E.E., Can C. (2025). Superiority of 68Ga-Trivehexin PET/CT Over 18F-FDG PET/CT in the Evaluation of Lymph Nodes in Patients With Breast Cancer. Clin. Nucl. Med..

[49] Novruzov F., Mehdi E., Aliyeva N., Orucova P., Simecek J., Aliyev J. (2025). The True Negative [^68^Ga]Ga-Trivehexin PET/CT in Solid Pseudopapillary Neoplasm of Pancreas, Mimicking Pancreatic Adenocarcinoma in [^18^F]FDG and [^68^Ga]Ga-FAPI Scans. Eur. J. Nucl. Med. Mol. Imaging.

[50] Emerson R., Agrawal K., Singh P., Patro P.S.S., Kumar N. (2025). Cancer Integrin Imaging With 68Ga-Trivehexin PET-CT in Head and Neck Squamous Cell Carcinoma Improves Diagnostic Accuracy. Clin. Nucl. Med..

[51] Wu H., Li L., Xiao Z., Chen Q., Li C., He Y. (2025). [68Ga]Ga-Trivehexin PET/CT Imaging of Integrin-Avβ6 Expression in Concomitant Mucinous Lung Adenocarcinoma and Idiopathic Pulmonary Fibrosis. Eur. J. Nucl. Med. Mol. Imaging.

[52] Moss B.J., Ryter S.W., Rosas I.O. (2022). Pathogenic Mechanisms Underlying Idiopathic Pulmonary Fibrosis. Annu. Rev. Pathol. Mech. Dis..

[53] Kuyumcu S., Denizmen Zorba D., Özkan Z.G. (2025). 68Ga-Trivehexin PET/CT Uptake in Malignant and Fibrotic Lung Tissue: Refining Diagnostic Applications. Eur. J. Nucl. Med. Mol. Imaging.

[54] Richeldi L., du Bois R.M., Raghu G., Azuma A., Brown K.K., Costabel U., Cottin V., Flaherty K.R., Hansell D.M., Inoue Y. (2014). Efficacy and Safety of Nintedanib in Idiopathic Pulmonary Fibrosis. N. Engl. J. Med..

[55] Mapelli P., Busnardo E., Magnani P., Freschi M., Picchio M., Gianolli L., Messa C. (2012). Incidental Finding of Parathyroid Adenoma with 11C-Choline PET/CT. Clin. Nucl. Med..

[56] Kuyumcu S., Denizmen D., Has-Simsek D., Poyanli A., Uzum A.K., Buyukkaya F., Isik E.G., Onder S., Aksakal N., Ozkan Z.G. (2024). 68Ga-Trivehexin PET/CT: A Promising Novel Tracer for Primary Hyperparathyroidism. Eur. J. Nucl. Med. Mol. Imaging.

[57] Treglia G., Piccardo A., Paone G., Trimboli P., Imperiale A. (2024). [18F]Fluorocholine PET/CT as First-Line vs. Second-Line Imaging Method to Localize Parathyroid Adenomas in Primary Hyperparathyroidism: “Game, Set, and Match.” Eur. J. Nucl. Med. Mol. Imaging.

[58] Caldarella C., De Risi M., Massaccesi M., Miccichè F., Bussu F., Galli J., Rufini V., Leccisotti L. (2024). Role of 18F-FDG PET/CT in Head and Neck Squamous Cell Carcinoma: Current Evidence and Innovative Applications. Cancers.

[59] Murakami K. (2011). FDG-PET for Hepatobiliary and Pancreatic Cancer: Advances and Current Limitations. World J. Clin. Oncol..

[60] Wu L., Hu J., Hua J., Liu M., Chen J., Xu J. (2012). Diagnostic Value of Diffusion-weighted Magnetic Resonance Imaging Compared with Fluorodeoxyglucose Positron Emission Tomography/Computed Tomography for Pancreatic Malignancy: A Meta-analysis Using a Hierarchical Regression Model. J. Gastro. Hepatol..

[61] Wang L., Dong P., Wang W.G., Tian B.L. (2017). Positron Emission Tomography Modalities Prevent Futile Radical Resection of Pancreatic Cancer: A Meta-Analysis. Int. J. Surg..

[62] Conroy T., Pfeiffer P., Vilgrain V., Lamarca A., Seufferlein T., O’Reilly E.M., Hackert T., Golan T., Prager G., Haustermans K. (2023). Pancreatic Cancer: ESMO Clinical Practice Guideline for Diagnosis, Treatment and Follow-Up. Ann. Oncol..

[63] Lu Q., El-Hashash A.H.K. (2019). Cell-Based Therapy for Idiopathic Pulmonary Fibrosis. Stem Cell Investig..

[64] Martinez F.J., Chisholm A., Collard H.R., Flaherty K.R., Myers J., Raghu G., Walsh S.L.F., White E.S., Richeldi L. (2017). The Diagnosis of Idiopathic Pulmonary Fibrosis: Current and Future Approaches. Lancet Respir. Med..

